# Human Leukocyte Antigen B*27-Negative Spondyloarthritis: Clinical, Serological, and Radiological Features of a Single-Center Cohort

**DOI:** 10.3390/diagnostics13233550

**Published:** 2023-11-28

**Authors:** Francesco Maria Mariani, Alessia Alunno, Evy Di Ruscio, Piera Altieri, Claudio Ferri, Francesco Carubbi

**Affiliations:** Internal Medicine and Nephrology Division, ASL1 Avezzano-Sulmona-L’Aquila, Department of Clinical Medicine, Life, Health & Environmental Sciences, University of L’Aquila, 67100 L’Aquila, Italyalessia.alunno82@gmail.com (A.A.); piera199413@gmail.com (P.A.); francescocarubbi@libero.it (F.C.)

**Keywords:** spondyloarthritis, human leukocyte antigen, ASAS classification criteria

## Abstract

The strong genetic association between HLA-B*27 and spondyloarthritis (SpA) accounts for about 90% of the susceptibility to axial SpA (axSpA), and the presence of HLA-B*27 is helpful in classifying patients according to the Assessment of SpondyloArthritis International Society (ASAS) classification criteria. However, over the years, other HLA-B alleles have been associated with an increased risk of developing SpA; on this basis, the aim of our study was to describe the demographic, clinical, and radiological characteristics of a cohort of SpA patients who were negative for HLA-B*27. We identified 85 patients with a clinical diagnosis of SpA displaying HLA-B alleles other than HLA-B*27; HLA-B*51 emerged as the most prevalent allele (N = 33, 39%), regardless of the fulfilment of either the axial or the peripheral ASAS criteria. The second most prevalent allele in the full cohort (N = 16, 19%) and in the patients fulfilling either the axial or the peripheral criteria was HLA-B*35. The third most prevalent allele in the full cohort was HLA-B*18 (N = 12, 15%), which was also the second most prevalent allele in the patients fulfilling neither of the two sets of criteria. Overall, the clinical picture was similar across the subgroups fulfilling the different sets of ASAS criteria; however, the patients not fulfilling any ASAS criteria had a higher likelihood of having arthritis compared to the patients fulfilling the axial criteria, whereas the Bath Ankylosing Spondylitis Functional Index was significantly higher in those patients fulfilling the axial criteria compared to those who did not fulfill any criteria. Our results indicate that other HLA alleles, beyond HLA-B*27, could be useful in facilitating SpA diagnosis, particularly in patients with a clinical picture which is consistent with SpA but does not fulfill the ASAS classification criteria.

## 1. Introduction

The term spondyloarthritis (SpA) encompasses a diverse array of chronic inflammatory disorders characterized by inflammatory back pain (IBP), asymmetric peripheral arthritis, enthesitis, dactylitis, and tenosynovitis. The clinical picture often also includes extra-articular manifestations (EAMs), like uveitis, psoriasis (PsO), and intestinal bowel diseases (IBD) [1,2]. Acute anterior uveitis occurs in 25–30% of patients with SpA and can be the first presenting symptom of the disease. Psoriasis develops in 10–25% of patients with SpA and IBD in only 5–10%. These disorders are typically characterized by the presence of the human leukocyte antigen (HLA)-B*27 allele and by the absence of the rheumatoid factor (RF) [2,3,4]. The strong genetic association between HLA-B*27 and SpA accounts for about 90% of susceptibility to axial spondyloarthritis (axSpA) [5], and the presence of HLA-B*27 is helpful in classifying patients as axSpA and peripheral SpA (pSpA) according to the Assessment of SpondyloArthritis International Society (ASAS) classification criteria [6,7]. The distinction between radiographic (r) and non-radiographic (nr) forms of SpA is closely tied to the evidence of sacroiliitis on a plain X-ray, a condition that constitutes one of the modified New York criteria and is therefore required for disease classification [8]. Both of these conditions fall within the predominantly axial clinical framework and are consequently included within the definition of ax-SpA. Conversely, if inflammation and pain primarily affect peripheral joints, sparing the sacroiliac joints, then we are dealing with a predominantly peripheral clinical presentation. These characteristics predominantly encompass lower limb and/or asymmetric arthritis, enthesitis, and dactylitis [7]; r-axSpA mostly affects young males during their productive, working years and is associated with chronic symptoms of pain, stiffness, and fatigue, leading to an impaired quality of life and frequent disability. Conversely, nr-axSpA seems more prevalent in woman than in men and has the burden of a longer diagnostic delay [9]. Although several aspects of SpA etiology and pathogenesis have been clarified, many aspects remain partially or totally unknown and intense research is taking place in this area [10]. Over time, a strong link between genetic predisposition and environmental factors has been demonstrated in several rheumatic diseases, and the Class I genes within the HLA system, such as HLA-A, HLA-B, and HLA-C, and the Class II genes, such as HLA-DQ, HLA-DR, and HLA-DP, all of which belong to the major histocompatibility complex (MHC), have been identified as promoters of these conditions [11,12]. Important environmental influences in SpA include gut microbial dysbiosis and entheseal stress or trauma [1]. In SpA, the frequency of the HLA-B*27 gene is 75–90% in both radiographic (r)-axSpA and non-radiographic (nr)-axSpA [13,14] and 27–47% in pSpA. However, only 5–8% of HLA-B*27-positive individuals develop SpA [15]. The geoepidemiology of SpA mirrors the carrier status of HLA-B*27 alleles in populations of different ancestries, with r-axSpA being less common in Japanese and African people compared to Chinese people and Caucasians [16]. In addition to HLA-B*27, over the years other HLA-B alleles have been associated with an increased risk of developing SpA, particularly axSpA, including HLA-B*13:02, HLA-B*40:01, HLA-B*40:02, HLA-B*47:01, and HLA-B*51:01, whereas the HLA-B*07:02 and HLA-B*57:01 alleles seem to be protective [17,18]. Likewise, other HLA-B alleles are associated with an increased risk of developing pSpA. Among these, HLA-B*15 [19], HLA-B*38 and HLA-B*39 [20] are limited to psoriatic arthritis (PsA), while HLA-B*35 and HLA-B*44 are essentially limited to IBD-SpA [2,21,22,23]. The aim of this study was to describe the demographic, clinical, and radiological characteristics of a cohort of SpA patients who were negative for HLA-B*27 and to find associations with other HLA-B alleles.

## 2. Materials and Methods

### 2.1. Patient Cohort

A retrospective study was conducted, recruiting consecutive patients with a clinical diagnosis of axial or peripheral SpA; the patients were tested for the HLA-B gene and were referred to our Immuno-Rheumatology service of the Internal Medicine and Nephrology Division at the University of L’Aquila, Italy, between November 2021 and August 2023. The HLA-B gene characterization was performed using the SSO Luminex technology [24]. HLA-B*27-negative patients fulfilling the classification criteria for other rheumatic and musculoskeletal diseases (e.g., Behçet disease) were excluded. The collected data included: age, sex, age at diagnosis, disease duration, the time frame between symptom onset and disease diagnosis, musculoskeletal clinical picture at onset, skin and mucosal manifestations (e.g., oral ulcers, genital ulcers, and PsO), ocular (e.g., uveitis) and gastrointestinal (e.g., IBD) involvement, and an increased C-reactive protein (CRP) value (> 0.5 mg/dL). Disease activity was evaluated using the Ankylosing Spondylitis Disease Activity Score (ASDAS)-CRP [25], and the Bath Ankylosing Spondylitis Disease Activity Index (BASDAI) [26]. The number of tender and swollen joints and the history of dactylitis were also recorded, whereas entheseal involvement was measured with the Maastricht Ankylosing Spondylitis Enthesitis Score (MASES) [27] and the Leeds Enthesitis Index (LEI) [28]. The following patient-reported outcomes (PROs) [29] were also collected: the Bath Ankylosing Spondylitis Functional Index (BASFI) [30], the Health Assessment Questionnaire (HAQ) [31], the Patient Global Assessment (PGA) [32], and the Visual Analogue Scale (VAS) of pain [33]. The patients with axial manifestations underwent X-ray and/or magnetic resonance imaging (MRI) in order to evaluate sacroiliac joints. Other variables, such as family history of SpA, ethnicity, comorbidities (including fibromyalgia), smoking habits, and menopausal status were also collected. 

All the methods were carried out in accordance with the Good Clinical Practice guidelines, and all the patients provided written informed consent in accordance with the Declaration of Helsinki. Ethical review and approval (by the internal review board of the University of L’Aquila) were obtained in accordance with local legislation and institutional requirements. 

### 2.2. Statistical Analysis

The data were analyzed with IBM SPSS 28.0 software, and the variables were compared using the chi-square test, the Mann–Whitney U test or the Kruskal–Wallis test with multiple post hoc comparisons. Univariate logistic regression was also performed. All the tests were two-tailed, and values of *p* < 0.05 were considered significant.

## 3. Results

We identified 85 patients with a clinical diagnosis of SpA displaying HLA-B alleles other than HLA-B*27. The demographic and clinical features are shown in Table 1. 

In our cohort, the majority of the patients (78%) were females with a mean age of 59.7 years, a mean disease duration of 3.7 years, and a mean gap between symptom onset and SpA diagnosis of 2.5 years. Seventeen patients (20%) fulfilled the ASAS classification criteria for peripheral SpA, 31 (36%) patients fulfilled the criteria for axial SpA, and 37 (43%) patients fulfilled neither of the two sets of criteria. A family history of SpA was reported by only 16% of patients. Forty-five patients reported IBP at disease onset and therefore underwent imaging assessment, and only 11 patients displayed sacroiliac involvement on an X-ray. 

Finally, in 32 (38%) of the 85 patients, CRP was above the upper normal level (0.5 mg/dl) at disease onset. 

Table 2 displays the distribution of HLA-B alleles in the study cohort. HLA-B*51 emerged as the most prevalent allele and was detected in 33 (39%) patients, followed by HLA-B*35 (N = 16, 19%), HLA-B*18 (N = 12, 15%), HLA-B*44 (N = 9, 11%), and HLA-B*13 (N = 9, 11%). The most frequent genotypes were HLA-B*51/HLA-B*18 and were observed in six (7%) patients, followed by HLA-B*51/HLA-B*07 and HLA-B*51/HLA-B*52, which were observed in three (9%) patients each. All the other genotypes were shown by one or two patients each. 

We then divided the 85 patients according to the fulfilment of the ASAS criteria (axial, peripheral, or neither of the two) [6,7] (Table 3). 

Females were equally represented across the three groups, and the patients displayed a similar age, age at SpA diagnosis, and time gap between symptom onset and SpA diagnosis. The disease activity scores, namely the BASDAI and ASDAS, were comparable between the three groups, and a similar behavior was observed for the PROs, such as the VAS pain and PGA. 

With regard to the functional activity indicators, the HAQ did not exhibit any significant differences between the various groups, while the BASFI was significantly higher in the patients who met the axial criteria as opposed to those who did not meet any criteria (*p* < 0.05). IBP with proven sacroiliac involvement on imaging was the dominant feature of the patients fulfilling the axial criteria. The prevalence of IBP was significantly lower in the patients not fulfilling any ASAS criteria and in those fulfilling the peripheral criteria compared to those fulfilling the axial criteria. Furthermore, the patients not fulfilling any ASAS criteria had the same likelihood of showing IBP compared to the patients fulfilling the peripheral criteria (OR = 0.4; 95% CI: 0.1–1.2), and sacroiliac involvement on imaging (X-ray or MRI) was detected only in the patients fulfilling the axial criteria. 

The patients not fulfilling any ASAS criteria also had a higher likelihood of having arthritis compared to the patients fulfilling the axial criteria (OR = 8.9; 95% CI: 2.7–29), whereas the likelihood of showing enthesitis was not different across the three groups. With regard to psoriasis, the patients fulfilling the peripheral criteria and those fulfilling the axial criteria showed it more frequently than the patients not fulfilling any ASAS criteria (OR = 15; 95% CI:1.6–141 and OR= 8.6; 95% CI: 1–76). 

In the patients fulfilling the ASAS criteria for axial involvement, the three most prevalent alleles were HLA-B*51 (N = 10, 32%), HLA-B*35 (N = 8, 26%), HLA-B*44, and HLA-B*07 (both N = 5, 16%) (Table 4). 

In the patients the fulfilling the ASAS criteria for peripheral involvement, the three most prevalent alleles were HLA-B*51 (N = 5, 29%), HLA-B*35 (N = 4, 24%), and HLA-B*18 (N = 3, 18%). Finally, in the patients fulfilling neither of the two sets of criteria, the three most prevalent alleles were HLA-B*51 (N = 18, 49%), HLA-B*18 (N = 7, 21%), and HLA-B*13 (N = 6, 16%). In the patients not fulfilling the ASAS criteria, the most frequent genotype was HLA-B*51/HLA-B*18 (N = 6, 16%), but these patients had a similar clinical picture compared to those showing other alleles.

## 4. Discussion

HLA-B*27 is the genetic hallmark of SpA, and its presence is helpful in classifying patients as axSpA or pSpA according to the ASAS classification criteria [6,7]. However, although HLA-B*27 can be observed in up to 90% of patients with either nr- or r-ax-SpA, not all SpA patients display HLA-B*27, and not all the mechanisms by which HLA-B*27 is involved in SpA pathogenesis are fully understood. In addition, the evidence showing that other HLA-B alleles are significantly associated with SpA HLA-B*27-negative individuals has accrued (Table 5) [34]. 

Our study aimed to describe the HLA alleles expressed by patients with a clinical diagnosis of SpA that lacked the HLA-B*27 allele. In particular, our results in a cohort of 85 Italian patients indicate that many other HLA alleles, beyond HLA-B*27, could be detected in the patients with a clinical picture consistent with SpA, regardless of the fulfilment of the ASAS classification criteria. In our cohort, twenty-eight HLA-B alleles other than HLA-B*27 were detected, and among these, HLA-B*51 was the most frequently observed allele in our cohort, regardless of the fulfilment of the ASAS criteria. In addition to HLA-B*51, the other alleles most frequently expressed were HLA-B*35, HLA-B*18, HLA-B*44, and HLA-B*13. Our observation on the prevalence of HLA-B*51 is in line with existing case reports [39,40,41,42] and cohort studies [43,44,45,46,47,48], which show that although HLA-B*51 remains the most important genetic factor in Behçet’s disease (BD) [36], it could also be associated with SpA. As far as HLA-B*35 is concerned, although this was the second most common allele that we observed in the patients fulfilling the axial and peripheral ASAS criteria, it was observed more rarely in patients not fulfilling these criteria. When exploring previously published data on HLA-B*35, we identified a study showing that this allele was associated with MRI sacroiliac involvement in HLA-B*27-negative patients with IBP and a clinical diagnosis of undifferentiated SpA (uSpA) [37]. In our cohort, six HLA-B*35-positive patients who fulfilled the axial ASAS criteria showed MRI involvement of the sacroiliac joints. Furthermore, two out of six patients also showed the same involvement on the X-ray assessment, suggesting irreversible damage. Of interest, in previous studies HLA-B*35 has also been associated with IBD-SpA [21,22,23]. In our cohort, the prevalence of IBD was very low and neither of the two patients with IBD showed HLA-B*35. HLA-B*18 was the third most common allele both in the patients fulfilling axial criteria and in those not-fulfilling any ASAS criteria. Conversely, HLA-B*18 was observed less frequently in the patients fulfilling the peripheral ASAS criteria. Interestingly, HLA-B*18 has been predominantly detected in patients with PsA characterized by axial involvement [38]. However, in our cohort only 1 out of the 12 patients with HLA-B*18 had PsO. HLA-B*44 and HLA-B*13 alleles exhibited the same prevalence in our cohort. HLA-B*44 has mainly been acknowledged for its link with IBD-related SpA [21,22,23] and, particularly, with IBD-associated peripheral polyarthritis. As mentioned above, the prevalence of IBD in our cohort was very small; hence, this association was not observed. Conversely, in our cohort HLA-B*44 displayed a distinct predominance in the patients fulfilling the axial criteria. With regard to HLA-B*13, the currently available data were derived from studies that mainly recruited patients with r-ax-SpA. As the full SpA spectrum was included in our cohort, it is not surprising that our data do not align with the existing literature. In particular, the previous studies showed a consistent expression on the HLA-B*13 allele in patients with r-ax-SpA, whereas in our cohort HLA-B*13 was more prevalent among the patients who did not meet any of the ASAS criteria [18]. Likewise, HLA-B*07 was previously found to be associated with a reduced risk of r-ax-SpA in a study cohort with 9′069 r-ax-SpA cases and 13′578 controls of European ancestry [17], whereas in our cohort it resulted in being one of the most prevalent alleles in patients fulfilling the axial criteria. As far as the clinical picture is concerned, we observed some interesting differences. In more detail, patients not fulfilling the ASAS criteria showed a significantly lower probability of having IBP compared to those with axSpA and a similar probability compared to those fulfilling the pSpA criteria. Although our data should be interpreted with caution due to the small sample size, they seem to suggest that patients not fulfilling the ASAS criteria show a predominantly peripheral presentation more often. However, it is worth emphasizing that the peripheral predominance within the cohort not fulfilling the ASAS criteria may be related to the lack of the HLA-B*27 allele. Furthermore, although no differences in disease activity or disease duration emerged across the subgroups fulfilling or not fulfilling the ASAS criteria, we noticed a more pronounced functional impairment, namely higher BASFI scores, in patients with axSpA. This finding likely mirrors the natural course of axial disease and the fact that these patients already had radiographic evidence of sacroiliac involvement from either X-ray or MRI assessment at the disease onset. In fact, the BASFI values of the patients with IBP but no imaging evidence of sacroiliac involvement were much lower. Conversely, functional impairment, as measured by the HAQ, did not differ between those patients who fulfilled the ASAS criteria and those who did not. We acknowledge that our study displays some limitations, such as the retrospective design, the small sample size with individuals of the same ethnicity (all of them where Caucasian), and an uneven female representation. Therefore, our results should be interpreted with caution and confirmed by larger studies. 

## 5. Conclusions

In conclusion, we described the demographic, clinical, and radiological features of Caucasian patients with a clinical diagnosis of SpA that lacked the HLA-B*27 allele. It is tempting to speculate that the genetic diversity highlighted by the expression of various HLA-B alleles may contribute to the variability in SpA clinical presentation and disease progression. Further investigation into larger cohorts of patients of different ethnicities and across the full SpA spectrum will shed additional light on this topic and possibly lead to the future inclusion of other HLA alleles as classification criteria. 

## Figures and Tables

**Table 1:** Demographic and clinical characteristics of the study cohort (85 patients).

	Mean	SD
Age, year	59.7	11.9
Age at diagnosis, years	56.1	12.2
Disease duration, years	3.7	3.4
Time gap of symptom onset–diagnosis, years	2.5	4.0
VAS pain	6.7	1.6
HAQ	1.2	0.5
PGA	6.0	1.6
BASDAI	5.1	1.7
BASFI	4.4	2.0
ASDAS	3.0	0.8
MASES	1.3	1.5
LEI	0.9	0.9
	**N**	**%**
Females	66	78
Peripheral SpA ASAS criteria	17	20
Axial SpA ASAS criteria	31	36
No criteria	37	43
Uveitis	0	0
Oral aphthous	2	2
IBD	2	2
PsO	12	14
Inflammatory back pain	45	53
Sacroiliac involvement on X-ray	11/45	24
Sacroiliac involvement on MRI	21/45	47
Arthritis	62	73
Enthesitis	35	41
Dactylitis	6	7
Family history of SpA	14	16
Fibromyalgia	12	14
Increased CRP	32	38

VAS, Visual Analogue Scale; HAQ, Health Assessment Questionnaire; PGA, Patient Global Assessment; BASDAI, Bath Ankylosing Spondylitis Disease Activity Index; BASFI, Bath Ankylosing Spondylitis Functional Index; ASDAS, the Ankylosing Spondylitis Disease Activity Score; IBD, inflammatory bowel disease; PsO, psoriasis; MRI, magnetic resonance imaging; CRP, C-reactive protein; MASES, Maastricht Ankylosing Spondylitis Enthesitis Score; LEI, Leeds Enthesitis Index; ASAS, Assessment of SpondyloArthritis International Society; SpA, spondyloarthritis.

**Table 2:** Distribution of individual HLA-B alleles in the overall cohort.

	N	%
HLA-B*51	33	39
HLA-B*35	16	19
HLA-B*18	12	15
HLA-B*44	9	11
HLA-B*13	9	11
HLA-B*07	8	9
HLA-B*15	8	9
HLA-B*50	7	8
HLA-B*57	6	7
HLA-B*14	5	6
HLA-B*37	5	6
HLA-B*08	4	5
HLA-B*49	4	5
HLA-B*52	4	5
HLA-B*38	4	5
HLA-B*40	3	3
HLA-B*41	2	2
HLA-B*58	2	2
HLA-B*05	2	2
HLA-B*39	2	2
HLA-B*45	2	2
HLA-B*47	2	2
HLA-B*04	1	1
HLA-B*12	1	1
HLA-B*17	1	1
HLA-B*53	1	1
HLA-B*55	1	1
HLA-B*56	1	1

**Table 3:** Demographic and clinical characteristics of the study cohort according to the fulfilment of ASAS criteria.

	Fulfilling Peripheral Criteria N = 17		Fulfilling Axial Criteria N = 31		Fulfilling Neither Criteria N = 37		
	Mean	SD	Mean	SD	Mean	SD	*p* Value °
Age, year	64.2	10.5	58.4	10.8	58.7	13.0	0.10
Age at diagnosis, years	59.7	12.2	54.7	11.1	55.7	13.0	0.25
Disease duration, years	5.2	6.8	3.7	2.0	3	1.4	0.09
Time gap of symptom onset–diagnosis, years	1.1	1.2	3.1	5.3	2.3	3.5	0.18
VAS pain	6.9	1.5	6.7	1.7	6.4	1.6	0.39
HAQ	1.16	0.6	1.2	0.5	1.1	0.5	0.71
PGA	6.5	1.6	5.8	1.5	5.9	1.6	0.18
BASDAI	4.9	2.02	5.6	1.6	4.7	1.6	0.09
BASFI	4.3	2.3	5.1	1.8	3.8	1.9	0.02
ASDAS	3.3	1.1	3.0	0.7	2.9	0.8	0.21
MASES	2.0	1.5	1.2	1.7	0.6	0.9	0.13
LEI	1.0	0.8	0.8	0.9	1.1	0.9	0.49
	**N**	**%**	**N**	**%**	**N**	**%**	*p* Value *
Females	12	71	24	77	30	81	0.7
Uveitis	0	0	0	0	0	0	-
IBD	1	6	1	3	0	0	0.4
Oral aphthous	0	0	1	3	1	3	0.8
Genital aphthous	0	0	0	0	0	0	-
PsO	5	29	6	19	1	3	0.02
IBP	8	47	31	100	9	24	<0.001
Sacroiliac involvement X-ray	0	0	11	35	0	0	
Sacroiliac involvement MRI	0	0	21	68	0	0	
Arthritis	17	100	13	42	32	86	<0.001
Enthesitis	7	41	18	58	10	27	0.06
Dactylitis	0	0	2	6	4	11	0.37
Family history of SpA	5	29	7	23	2	5	
Increased CRP	10	59	9	29	13	35	0.17
Fibromyalgia	0	0	8	26	4	11	

VAS, Visual Analogue Scale; HAQ, Health Assessment Questionnaire; PGA, Patient Global Assessment; BASDAI, Bath Ankylosing Spondylitis Disease Activity Index; BASFI, Bath Ankylosing Spondylitis Functional Index; IBD, inflammatory bowel diseases; PsO, psoriasis; MRI, magnetic resonance imaging; CRP, C-reactive protein; MASES, Maastricht Ankylosing Spondylitis Enthesitis Score; LEI, Leeds Enthesitis Index; ASDAS, the Ankylosing Spondylitis Disease Activity Score. ° Calculated with the Kruskal–Wallis test comparing the 3 groups. * Calculated with the chi-square test comparing the frequencies in the 3 groups. Odds ratios resulting from the comparison of each group with the other using logistic regression analysis are reported in the manuscript.

**Table 4:** Distribution of individual HLA-B alleles according to the fulfilment of the ASAS criteria.

	All Patients N = 85	Fulfilling Peripheral Criteria N = 17		Fulfilling Axial Criteria N = 31		Fulfilling Neither Criteria N = 37	
	N	N	%	N	%	N	%
HLA-B*51	33	5	29	10	32	18	49
HLA-B*35	16	4	24	8	26	4	11
HLA-B*18	12	3	18	2	7	7	21
HLA-B*44	9	2	12	5	16	2	5
HLA-B*13	9	2	12	1	3	6	16
HLA-B*07	8	0	0	5	16	3	8

**Table 5:** Summary of available evidence about HLA alleles.

HLA	BD	uSPA	IBD-SpA	PsA	AS	AS (PROTECTIVE)
B*27					✔ [35]	
B*51	✔ [17,36]					
B*35		✔ [37]	✔ [21]			
B*18				✔ [38]		
B*44			✔ [23,37]			
B*13					✔ [17]	
B*07						✔ [17]

HLA, human leukocyte antigen; BD, Behçet’s disease; uSpA, undifferentiated spondyloarthritis; IBD, inflammatory bowel diseases (IBD); PsA, psoriatic arthritis; AS, ankylosing spondylitis.

## Data Availability

All relevant data are included in the manuscript.
